# “Exploring the relationship of organizational virtuousness, citizenship behavior, job performance, and combatting ostracism” through structural equational modeling

**DOI:** 10.1186/s40359-024-01873-9

**Published:** 2024-07-09

**Authors:** Eimad Hafeez Gogia, Zhen Shao, Karamat Khan, Mohd Ziaur Rehman, Hossam Haddad, Nidal Mahmoud Al-Ramahi

**Affiliations:** 1 School of Economics and Management, Harbin Institute of Technology, Harbin, Heilongjiang China; 2 School of Digital Commerce, Zhejiang Yuexiu University, Shaoxing, Zhejiang China; 3 Department of Finance, College of Business Administration, King Saud University, P.O. Box 71115, Riyadh, 11587 Saudi Arabia; 4 Zarqa University, Zarqa, 11831 Jordan; 5 Business Faculty - Accounting Department, Zarqa University, Zarqa, 11831 Jordan

**Keywords:** Organizational virtuousness, Organizational citizenship behavior, Job performance, Workplace ostracism, Banking Sector, SEM, Moderated Mediation

## Abstract

**Background:**

This theoretical model has been drawn on principles of social exchange theory to scrutinize the connection between organizational virtuousness and job performance with the mediating role of Organizational citizenship behavior and moderating role of workplace ostracism. A survey was conducted in Pakistan, gathering data from 486 employees working for various private and commercial banks.

**Method:**

Soft and hard questionnaires were distributed to the participants, with social media platforms used for the soft questionnaires and meetings with employees for the hard questionnaires. A 7-point Likert scale was employed in data collection, and measures for the variables were adapted from reliable and valid sources. A demographic analysis was performed to summarize the sample collected from participants. The demographics results were analyzed using SPSS, while the measurement model and path analysis were conducted using Structural Equational Modeling with Smart PLS-4.

**Results:**

The study’s findings showed a significant and positive relationship between organizational virtuousness and job performance, with organizational citizenship behavior serving as a mediator. Additionally, a negative moderation of workplace ostracism was observed in the mediation of organizational citizenship behavior toward the relationship between organizational virtuousness and job performance.

**Conclusion:**

The study’s results contribute to the implementation of social exchange theory and related concepts in the banking sector of Pakistan, providing practical guidance for implementing virtuous practices within organizations and discouraging ostracism in banks to enhance overall performance. The study suggests that policies regarding the implementation of virtuous practices in organizations can be established, and workplace ostracism can be avoided by providing a platform for social gatherings and training employees. Managers should adopt appropriate leadership styles and relevant communication patterns to impact the organizational climate which can also help reduce the influence of ostracism in the organization. Additionally, a complaint cell should be established with complete confidentiality to reduce ostracism.

**Supplementary Information:**

The online version contains supplementary material available at 10.1186/s40359-024-01873-9.

## Introduction

The banking sector of any country serves a valuable and significant part in the support of economic growth leading to economic development. In the uncertain economic condition of Pakistan with the less developed capital and money markets banking sector has to gain a significant position in the performance [1]. Due to terrorism and mistrust between the government and foreign donors, the fund’s inflow and foreign capital have been decreased and the only significant way of treatment is to rely on the banks to accommodate the country’s economy through the capital for different sectors of economic needs [2]. The world has faced unpredicted variations in the economic system position of the world due to the recent occurrence of COVID-19. Lower economic growth has been observed affecting the banking sector of developing countries by COVID-19 leading to uncertainty and reducing credit growth and foreign investment [3]. Different challenges for management have been provided in these prevailing conditions and it is difficult to find ways for profitability and economic growth in this uncontrollable external environment.

Unique strategies have to be obtained and implemented by the banking sector to tackle the aggressive competition and globalization in the markets to enhance their internal and external performance [4]. Employees’ job performance (JP) is an important contributor to the banking sector’s financial performance. Due to changes in the working environment, the definition of performance has been changed [5]. Employee performance is an imperative factor considered by organizations for the achievement of high organizational performance [6]. However, most banks are facing a significant decline in the performance of individuals [7]. For the sustainability and survival of the banks, it is necessary to have employees with high performance meeting the expectations of customers and higher management. Amid these challenges, the concept of Organizational Virtuousness (OV) becomes increasingly relevant. Scholars such as Cameron, Bright [8, 9], and Wright and Goodstein [10] have expressed their interest in the idea that cultivating virtues can lead to individual improvement and enhance organizational efficiency and performance.

In the organizational context, organizational virtuousness (OV) refers to the practice, support, dissemination, perpetuation, and nurturing of different dimensions of virtues such as forgiveness, integrity, humanity, and trust through individualistic or collectivist approaches [8]. Limited attention to the virtuous practices in the organization has been received in the existing literature and has not been implemented prominently in the organizations. Nonetheless, the recent worldwide incidents have caused disruptions to both financial and ethical aspects, which have drawn the interest of various stakeholders, including the business world, mainstream media, and trade publications. Consequently, researchers and managers have come to appreciate the advantages of cultivating virtue at both the individual and organizational levels. One noteworthy and recent example is a study conducted by Meyer [11], which explored the influence of various organizational virtuousness (OV) concepts on both organizational performance and employee behavior. The research findings revealed a positive impact of OV, with clear associations with improved financial and economic aspects of organizations. Equally significant was the enhancement in the ethical behavior of employees, demonstrating the multifaceted advantages associated with the cultivation of OV within organizational contexts. In this study the mediating impact of OCB has been checked in the relationship of OV and JP.

Organizational citizenship behavior (OCB) can play a potential role of mediation in the organizational virtuousness (OV) and employee job performance (JP) relationship. Organizational virtuousness entails the cultivation of virtuous values and practices within an organization, emphasizing ethical conduct, empathy, and a commitment to the well-being of all stakeholders [8]. Workers who hold a favorable view of their organization’s virtuous behavior are generally more driven and invested in their work [12]. The ground for the encouraged behaviors like virtuous practices can be set by organizational culture. Cultures valuing accountability, fairness, and transparency are about virtuous organizations that encourage ethical decision-making by employers. Virtuous culture can reinforce organizational culture and vice versa. Moreover, virtuousness can lead to positive outcomes like performance and satisfaction because it fosters commitment, trust, and cooperation which are the important elements of a positive organizational culture. This positive organizational culture can, in turn, encourage employees to exhibit OCB, which entails going beyond their formal job responsibilities to aid coworkers, support the mission of the organization, and contribute to a harmonious work environment. Organizational culture has a significant impact on the effectiveness of management in getting good outcomes [13]. One of the strongest elements in exhibiting OCB is the strong organizational culture which can be generated with a strong perception about the organizational or supervisor support. The impact of OCB on worker performance can be substantial [14]. Employees who engage in OCB often exhibit higher levels of commitment, teamwork, dedication, and hence performance in their roles. They are more likely to collaborate effectively, share knowledge, and proactively solve problems, ultimately leading to improved job performance. OCB can also enhance overall organizational effectiveness by fostering a positive workplace atmosphere and improving relationships among team members [15].

Several researchers also pointed out the significant role of workplace ostracism as a moderator in the context of OV and employee JP. Workplace ostracism (WO) refers to a form of social exclusion or isolation that occurs within an organizational setting [16]. It involves individuals or groups intentionally ignoring, excluding, or marginalizing one or more employees, making them feel isolated, unimportant, or invisible within their workplace community. Workplace ostracism often manifests through subtle behaviors, such as ignoring someone in meetings, excluding them from social gatherings, or withholding important information and communication [17]. This exclusionary behavior can have damaging effects on the target’s well-being, job satisfaction, and overall workplace performance, creating a toxic and emotionally distressing work environment [18]. There is a disturbance in the physiological and psychological health of employees due to stress at work can have the worst impact on employee health [19]. When employees experience ostracism, they may perceive a lack of fairness, trust, and respect within the organization, which can undermine their morale and motivation. Employees facing ostracism are less inclined to fully participate in their tasks [20], even in a virtuous organizational culture. Ostracism can erode employees’ commitment to the organization’s virtuous values and practices. OV often encourages collaboration and teamwork. Ostracism can impede employees’ willingness to collaborate with their colleagues, impacting collective performance [16]. Workplace ostracism can negatively affect employees’ psychological well-being, leading to stress, anxiety, and decreased job satisfaction [21]. In summary, the presence of WO can moderate the relationship between OV (organizational virtuousness) and employee JP (job performance) by introducing negative factors that counteract the positive effects of virtuousness.

This study enhances the comprehension of how OV, JP, OCB, and WO are interconnected. There are numerous prominent gaps in the existing literature that this study addresses. First, prior research on organizational virtuousness and its impact on job performance is relatively scarce, particularly within the framework of the banking network of Pakistan. This study fills a gap by exploring this relationship, delivering important knowledge for academics and practitioners alike. Furthermore, the introduction of organizational citizenship behavior as a mediating variable adds depth to the understanding of how virtuousness influences job performance. This insight can inform HR practices and organizational culture development. Finally, the identification of workplace ostracism as a moderating factor is a noteworthy contribution. Recognizing the harmful influence of ostracism on the mediation process underscores the need for organizations to address and mitigate such negative workplace behaviors.

The main purpose of this study done in Pakistan is that people are deeply connected, interdependent, and loyal to each other in an inner circle-like organization which is called collectivism. Most of the organizations in Pakistan are working under a collectivist culture and hierarchical organizational structure in which respecting the authority and social norms can exclude the people not accepting the norms [22]. Different studies show comparatively higher incidents of ostracism in the workplace than the organizations focusing on individualism [23]. Most of the complex interactions between different organizations and employee performance have been studied in the banking sector. There is diversity in the banks regarding responsibilities, job roles, and employee engagement making an ideal situation for observing these variables. The influence of virtuous practices on employee performance and OCB can significantly impact the organizational reputation through customer satisfaction which is important for the financial institutions and economic growth. There have been significant transformations and challenges like technological upgradation, regulatory changes, and economic shifts recently. Therefore the banks in Pakistan offer a unique and relevant opportunity for the investigation of these variables.

Organizations are facing moral dilemmas whether it’s the educational institutions or the corporate sector and ostracism in the workplace is one of them. The moral difficulties in the organizations are pushing the business circle to focus on the ethics in the organizations [24] highlighting the importance of virtuous practices in the workplace [10]. The relationship between OV and JP with the mediating role of OCB and moderating role of workplace ostracism can be seen through the lens of social exchange theory (SET) which shows that social behaviors can be the results of the exchange process by the organization. The main objective of all exchanges within the organization is to minimize costs and maximize benefits. SET elaborates that when the employee’s perception of the virtuous practice is high, they will be engaged in social behaviors like OCB. However perceived benefits and social cost of engaging in OCB can be outweighed by altering the exchange balance leading to reduced performance.

The remaining sections of this study following the introduction are the literature review in the second section with hypotheses development, methodology, and findings in the third section with the next section having a conclusion and discussion, theoretical and practical implications. At last limitations and recommendations for future research have been elaborated.

## Theoretical foundation, literature, and hypothesis

### Social exchange theory

Social exchange theory (SET), as formulated by Richard and Emerson [25], provides a valuable theoretical and analytical framework for understanding the exchange of resources and the dynamics of social interactions. This theory encompasses both tangible and intangible exchanges between individuals or parties [26]. In the context of this study, we explore how organizations engage in reciprocal actions, such as OCB and JP, as virtuous exchanges. The model in this article has been underpinned by social exchange theory with the explanation of how positive behaviors (OCB and Virtuous climate) and negative (WO) interact with each other influencing job performance. Feelings of obligations with trust can be fostered by positive exchanges while ostracism at the workplace may erode these feelings reducing positive organizational behaviors and effectiveness [27]. The fundamental aspect of social exchange theory is reciprocity elaborating that OCB can be a useful and critical pathway through which employee job performance can be impacted by organizational virtuousness. Employees respond positively to virtuous actions within their workplace by aligning their behaviors [28]. This alignment of behavior can be attributed to the concept of social exchange, where individuals reciprocate in response to received benefits [28]. It is noteworthy that virtuousness in this context is not solely driven by individual self-interest but is also concerned with the well-being of others, contributing to a sense of moral goodness and social benefits [29]. SET underscores that exchange processes motivate workers to engage in pro-organizational behaviors and attitudes [30].

In a workplace context, job performance is a critical outcome that must be carried out efficiently and effectively [31]. Derived from the social exchange concept, there is the perception of employees about the virtues that are beneficial for them which in turn stimulates them to desire to reciprocate it through enhanced job performance [32]. Workplace ostracism, as indicated by Ferris, Lian [33], is associated with inadequate employee performance. This aligns with the core ideas of social exchange theory. When employees experience ostracism, they may reduce their effort and engagement, resulting in suboptimal outcomes [34]. SET describes that positive exchanges can be undermined in the organization by ostracism can weaken the motivational impact of OCB on employee job performance [35]. Additionally, the withholding of vital information by employees, as seen in the study by Fatima, Bilal [36], is consistent with the SET framework. When employees feel ostracized, they may be less inclined to share crucial information necessary for both individual and organizational performance and growth. The mediating impact of OCB is stronger with the low ostracism at the workplace because employees are more likely to engage themselves in productive behaviors when they feel included and supported. Meanwhile, the mediating impact of OCB might be weakened by high levels of ostracism as positive influence can be counteracted by negative social exchanges [37].

In conclusion, SET provides a valuable framework for understanding how virtuous actions, reciprocation, and workplace ostracism influence organizational behaviors and outcomes. This theoretical perspective highlights the importance of fairness and equitable exchanges in driving positive attitudes and behaviors within the workplace.

### Organizational virtuousness and job performance

Organizational virtuousness (OV), though often overlooked by organizations in Pakistan, holds a crucial role, particularly during times of financial and moral crises. Recognized as a best practice, OV has the potential to enhance both individual and organizational efficiency and performance, making it a central agenda for organizational policies [38]. This concept encompasses individual and collective actions, as well as cultural attributes, that promote and sustain virtuousness within an organization [8]. Moreover, OV has a noteworthy impact on JP, as established in the study by [8], which also identified amplifying and buffering effects. These effects not only yield positive results but also safeguard employees from negative impacts.

The connection between OV and employee job performance has been well explained with the SET. SET posits the social interaction within the organization which is based on the reciprocity theory in which there is an exchange of support, resources, and trust. These exchanges are based on the social relationship of employees within the organization impacting the behavioral patterns of employees in the organization. In the previous research, Cameron, Bright [8] elaborated empirically on the positive relationship between OV and different outcomes like organizational commitment, and employee JP. Therefore the relationship between OV and JP can be analyzed through SET based on trust, social support, and reciprocity.

Numerous studies affirm the significant impact of OV on employee performance, indicating that when workers perceive their organization as caring, trustworthy, and respectful, they tend to perform their duties efficiently and effectively [39]. Furthermore, OV extends its positive influence to individual self-management, as it significantly enhances JP. Scholars have explored OV’s relationship with various positive job outcomes, such as OCB and commitment, ultimately showing its profound impact on employee job performance [40–42]. This research emphasis on JP, along with a more recent focus on organizational citizenship behavior, provides a complete consideration of the motivating effects of OV in organizational settings. In conjunction with the definition of JP as actions contributing to organizational goal achievement, the interrelation between OV and performance forms a crucial component of organizational dynamics [43]. Hence it can be hypothesized that:-.

*H1: Organizational virtuousness is significantly and positively related to employee job performance supported by social exchange theory.*

### Mediation of organizational citizenship behavior

Organizational Citizenship Behavior (OCB) can be defined as individual behavior utilized to enhance an organization’s effectiveness, and it is unrelated to the organizational reward system [44]. This involves employees going above and beyond the organization’s demands, contributing in a special way known as OCB [45]. OCB encompasses a range of behaviors such as assisting colleagues, volunteering for extra activities, and motivating others. OCB can be recognized as an acritical mediator in the relationship between OV and JP. Understanding these relationships offers valuable insights for organizations and managers aiming to enhance overall performance through a positive work environment. Many studies have underscored the importance of OCB as a vital function within organizations [46]. It provides an alternative perspective on performance [47]. An organization heavily relies on employees’ contributions beyond their assigned tasks, known as citizenship behavior [48]. Therefore, it is considered a cornerstone of organizational success [49]. On the other hand, performance is the outcome of the processes that employees have undertaken, or it can be quantified in terms of quality [50]. Therefore, performance is measurable primarily based on results, and it reflects how effectively employees have achieved organizational goals [51]. This underlines the crucial distinction between OCB, which is an employee’s extra-role contribution, and performance, which is primarily outcome-driven.

When employees perceive the virtuousness of their organization positively, it motivates them to exhibit positive behaviors and emotions, fostering employee engagement and their overall impression of the organization [52]. Employees perceiving virtuous practices from the organization are more likely to engage themselves in citizenship behavior motivated by intrinsic satisfaction [40]. These behaviors directly contribute to better cooperation, coordination, and a supportive working environment conducive to good performance. Employee loyalty and voluntary behavior can be enhanced to strengthen the positive image through the positive perception of OCB [53]. Ethical standards and moral climate can be enhanced by OV within the organization fostering integrity and trust. This positive climate and ethical practices encourage workers to engage in OCB driven by commitment and motivation towards organizational goals. In-turn OCB can improve employee job performance fostering cooperation and organizational efficiency and reducing conflicts.

The role of OV has been predicted in the citizenship behavior of employees with the support of SET in the study done by Mayer, Kuenzi [54]. Findings show a positive relationship among the variables. When the employees perceive positively the virtuous practices in the organization show good citizenship behavior in the organization. In another study, Wieseke, Ahearne [55] explained the findings of different studies showing the positive impact of Organizational Identification and perceived organizational support which is also an important element of OV on employee JP in the light of SET. Findings suggested that if there is a positive perception about the organization, there will be good performance by the employees.

It has been suggested in different research that OV helps induce positive outcomes through pro-organizational attitudes and behaviors. Employees facing positive organizational experiences related to different forms of virtuous practices mostly show helping behaviors towards colleagues, and clients, suggesting constructive points, and protecting the organization from external stakeholders [8]. In the context of OCB, five dimensions have been identified: altruism, courtesy, sportsmanship, conscientiousness, and civic virtue. Studying these extra-role behaviors and their impact on employee performance contributes significantly to organizational effectiveness [56]. Studies provided empirical evidence suggesting a positive relationship between OV and OCB [40]. Another stream of research has also examined the OCB as a mediator between leadership styles and individual job performance, confirming that OCB indeed plays a mediating role between ethical leadership and employee performance [57]. Overall, the existing literature reinforces the idea that OCB is a vital aspect of employee behavior and it significantly influence the individual and organizational performance and effectiveness. Hence it can be hypothesized that:-.

*H2: OCB is significantly and positively mediating the relationship between Organizational Virtuousness and job performance.*

### Moderation of workplace ostracism

Workplace ostracism (WO) refers to the feeling of being ignored by everyone in an organization [58] and can have significant negative effects on individual and organization performance. Employees who experience chronic rejection may suffer from issues like depression, isolation, and anxiety, which can hinder their ability to meet their goals and fulfill their needs [59]. Ostracism also reduces social communication, making it difficult for individuals to fulfill their psychological needs and negatively affecting their mental and physical health [60]. It can even cause employees to feel disconnected from the organization and its members [61].

Workplace ostracism is all about the exclusion and isolation of employees within the organization also having a detrimental impact on productivity, wellbeing, and overall organizational culture. HR practices should be aligned with the organizational interventions to handle the issue of WO. Training and awareness is one of the valuable HR practices that can recognize and handle WO problems. Empathy, diversity awareness, and communication skills should be included in the training and awareness program [62]. Transparent communication policies can be the second important step in handling WO. Regular meetings with employees and an anonymous suggestion box bridge the gap between employer and employee can reduce the chance of WO [63]. Moreover, the development and enforcement of a zero-tolerance policy with disciplinary measures against WO can help employees enhance their productivity and performance [64]. Team building creates a sense of belonging and camaraderie between workers helping the employer to reduce WO. Mutual support and collaboration in the volunteer projects and different organizational workshops is the key to promoting interpersonal relationships among employees which breaks down the barriers among workers [65].

Employees tend to act in line with their perception of the organization and are more likely to support their co-workers when the organization acknowledges their efforts or when employees positively perceive organizational virtuousness [40]. However, WO makes it challenging for senior management to implement positive practices and prevents employees from engaging in positive behaviors like OCB and JP. Therefore, when the needs of individuals suffering from workplace ostracism are ignored, it can lead to negative consequences such as retaliation and reluctance to share knowledge [60]. It can also result in counterproductive work behavior if employees feel ostracized within the organization [66].

The moderating role of WO has been examined before in many studies which will strongly justify the moderating role of WO in the relationship between OV and OCB. The impact of WO on OV and OCB can be moderated by organizational culture and leadership. Leadership can help employees accept the change quickly [67] and mitigate the impact of ostracism. Similarly, the detrimental effect of ostracism on discretionary efforts and virtuous behaviors can be buffered by the organizational culture which has been characterized by empathy, fairness, and inclusivity. Social affiliation and belongingness are necessary for every employee for a better outcome. As an individual everyone is seeking social affiliation, inclusion, and belongingness [68]. Most of the part of an individual is filled with interpersonal relationships and can have significant personal implications. Virtuous practices in the organization can help employees build interpersonal relationships within the workplace. The accomplishment of collective goals with a sense of support is due to the good relationships at the workplace [69]. However one of the ubiquitous elements in the workplace is ostracism. Some kind of ostracism has been faced by most of the employees in the workplace [70]. Emotional stress can be generated and a sense of belongingness can be hurt by workplace ostracism [69]. The most significant threat of workplace ostracism is the individual and organizational outcomes gaining much attention from scholars. There are behavioral consequences like OCB, workplace deviance, and job performance [69]. The negative relationship between WO and extra-role behaviour has been highlighted by O’Reilly, Robinson [71]. This also aligns with the study of Sahoo, Sia [72] and Mao, Liu [73] which shows that WO can lead to unfavorable work behaviors and attitudes.

The moderating role of ostracism can be studied in the light of SET which shows the reciprocal of social interactions. Focusing on workplace ostracism employees may have a perception of the breach in the social contract [74] and may show less OCB and performance experiencing a lack of fairness and reciprocity. In the previous study, Li, Xu [75] investigated the consequences of WO on the relationship between OV and employee job performance. The study revealed the negative impact of workplace ostracism on the outcomes and it can be mitigated through virtuous practices within an organization.

Different studies are providing empirical evidence of OCB, in the light of WO. In the study presented by Rong, Zheng-Rong [76] the relationship of WO with In-role and extra-role behaviors (OCB) has been found negative. In another study, the impact of co-worker ostracism has been checked on OCB with the moderating role of ethical leadership [77]. Findings show that ethical leadership can reduce the harmful impact of WO on OCB. Different studies are focusing on the detrimental impact of workplace outcomes like task performance and OCB [78, 79] The above studies provide empirical evidence of the negative influence of WO on OCB. Ostracized employees not only show negative performance but also show less citizenship behavior by employees further influencing organizational outcomes.

Recent studies show that there can be many negative consequences of WO, like low satisfaction, emotional exhaustion, reduction of prosocial behavior, low OCB, and an increase in deviant behaviors within the organization [80]. It has also been notable that WO and employee JP have a negative relationship [81]. Previously the impact of WO has been checked on the performance with the moderation of perceived organizational support and found significant results and a strong negative relationship among variables [82]. Some studies have shown the negative impact and moderation of WO in the way of employee and organizational outcomes. In the study done by Yang and Wei [83], the impact of ethical leadership has been checked on OCB with the mediating role of organizational commitment and the moderating role of WO. The findings elaborate that workplace ostracism mitigates the relationship between ethical leadership and OCB. In another study, the relationship between workplace spirituality has been checked on OCB with the mediation of organizational commitment and the moderating role of WO representing a mediated moderation relationship [84]. The study revealed that the relationship between workplace spirituality and OCB has been mediated by organizational commitment and the impact has been reduced by the moderating impact of WO. Most of the research shows showing negative impact of workplace ostracism. In the study done by Ametepe, Otuaga [85], the moderating impact of workplace ostracism has been checked in the relationship between workplace climate (employee participation and training) and organizational commitment. The finding revealed the negative moderation of workplace ostracism in the relationship between organizational climate and organizational commitment.

The intricate dynamics of the organizational settings are helpful for the development of the proposed mechanism in this study which shows the moderating role of WO on the mediation of OCB in the relationship of OV and JP. Research shows that the perception of OV among employees impacts engagement in OCB positively. When employees’ perception is positive about the virtuous practices within the organization, they may show positive behavior voluntarily which can contribute to the well-being of the organization [86]. Therefore OCB can play a significant role in the relationship between OV and JP.

WO can impact the employee’s behaviors and perceptions. Employees may feel less motivated, disengaged, and less committed when they feel ostracism in the workplace. The negative impact of WO may lead to a reduced OCB and they fail to establish and maintain better relationships with their colleagues [87]. Therefore WO attenuates the positive influence of OV and OCB and moderates the relationship.

On the relationship of OV-OCB, the moderating role of WO has implications for job performance. Ostracism diminishes the employee’s engagement in OCB and also affects their job performance. Less OCB means less contribution to the organizational functions and effectiveness leading to poor employee performance and other outcomes. Therefore it can concluded that there is a negative moderation of WO in the mediation of OCB in the relationship of OV and JP. Hence it can be hypothesized that:

*H3: Workplace ostracism significantly and negatively moderates the mediation of organizational citizenship behavior in the relationship between organizational virtuousness and job performance.*

**Fig. 1:**
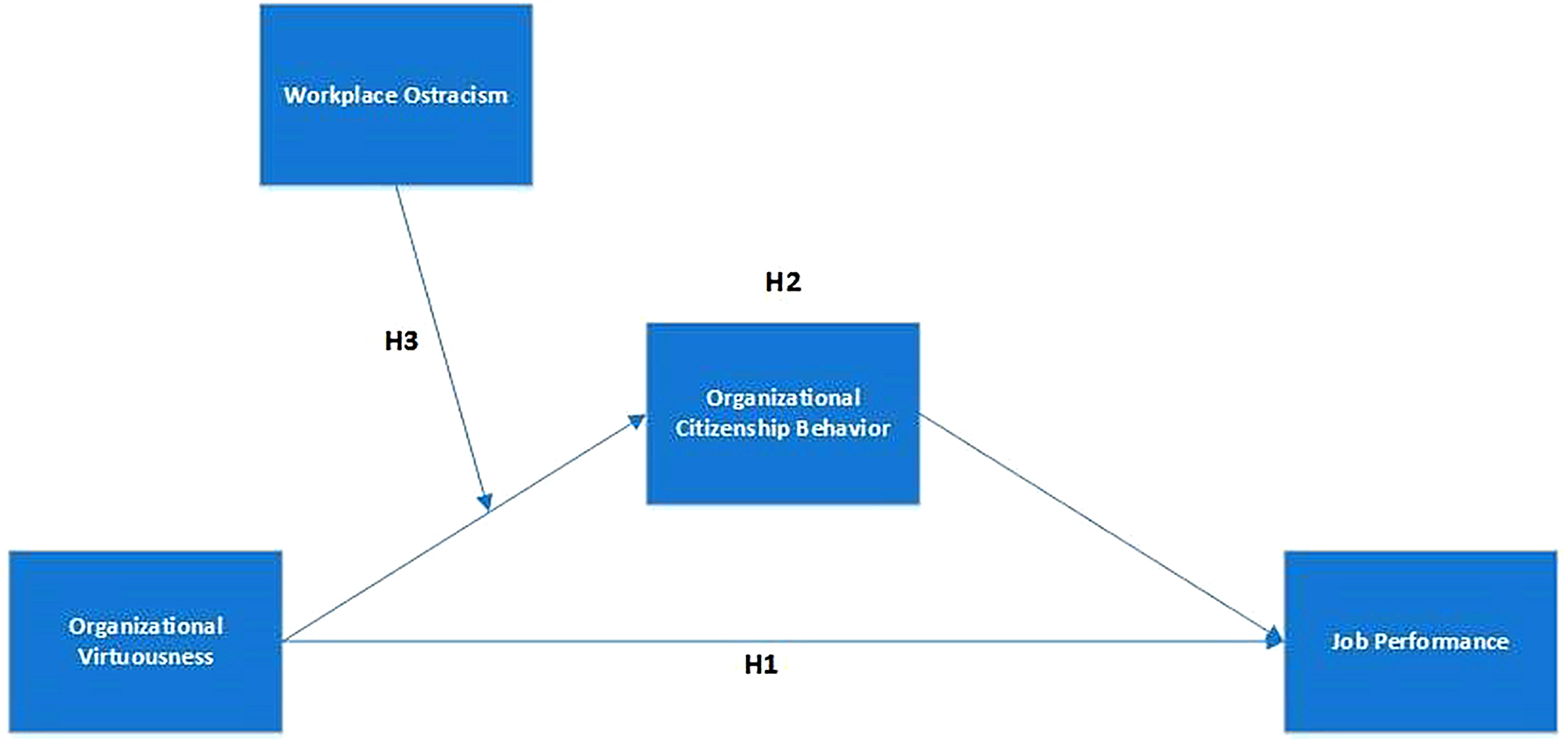
Conceptual framework

In the study conducted by Magnier-Watanabe, Uchida [38], researchers examined the impact of organizational virtuousness on employee job performance, considering subjective well-being as a mediating factor. The study also highlighted certain limitations and provided recommendations for future research. One such recommendation was to explore additional outcomes of organizational virtuousness, such as OCB. Building on this, the current framework introduces OCB as a mediator in the relationship between OV and JP, thereby adding novelty to the research. In another study by Arshad, Arshad [88], the effects of organizational virtuousness and emotional intelligence on in-role and extra-role performance were investigated, with work-related subjective well-being serving as a mediator. The authors suggested examining the role of moderators in the direct relationship. Addressing this, the current model introduces workplace ostracism as a moderator in the mediation process of OCB, particularly focusing on collectivist societies like Pakistan. This addition aims to bring new insights into the model. Furthermore, a study by Sun and Yoon [89] explored the impact of OV on OCB, with work engagement as a mediator and proactive personality and organizational support as moderators. However, this study was limited to Korean employees, raising questions about its generalizability. To address this limitation, the current research investigates the impact of organizational virtuousness on employee job performance, considering OCB as a mediator and workplace ostracism as a moderator, thereby expanding the scope of the research beyond the initial context (Fig. 1).

## Methodology and findings

### Research setting and data collection

Data for this study was collected through questionnaires distributed to bank employees working in various private and commercial banks in Pakistan. A 7-point Likert scale has been used in this study with the value 1 for strongly disagree to 7 for strongly agree. The questionnaire has been distributed by visiting different banks and using different social media platforms like Facebook, Linkedin, WhatsApp, etc. A soft copy of the questionnaire has been prepared in Google Forms and links have been shared with respondents. The printed questionnaires have been distributed to the employees. The participants were informed about responding to the questionnaire voluntarily. The valuable expertise of my references has been harnessed for the curation of comprehensive data collection ensuring the accuracy and depth of findings. Different banks have different social media groups of their employees through which the questionnaire can reach the broader population. Moreover, data confidentiality and privacy have been ensured by not asking for their name, designations, contact numbers, or emails in the questionnaire. Convenient sampling has been used in this study. The main reason for choosing the convenient sampling is because the population of interest was difficult to access and the sample has been taken from private and commercial banks of Pakistan. Moreover, convenience sampling is cost-effective in terms of resources and time. The other methods sometimes required planning and coordination and sometimes financial requirements to collect data from participants.

To minimize the biases in the data collection, data has been collected from different locations in Pakistan to focus on a broader range of perspectives. This also enhances the generalizability of the study. Moreover, data has been collected using different methods (social media and in-person) which also reduces biases in the study. Randomization has been used in collecting the data which has an equal chance of sample inclusion. Transparency has been ensured in the data collection which is also helpful in the reduction of biases.

A total of 510 responses were initially received, with invalid and incomplete responses subsequently removed, resulting in a final dataset of 486 valid responses. This sample size aligns with the guideline that suggests it should exceed the total number of items, given that this study includes 24 items in the questionnaire [90]. Additionally, the sample size adheres to the general rule of having 10 to 30 respondents on behalf of each independent variable [91]. The questionnaire comprises five different parts, with the first part focusing on the respondent’s demographics.

For more justification regarding sample selection, G-Power has been used for the sample size calculation. G power analysis is significant by using multiple predictors. In this model, the balancing power of 0.80 has been used with the significant level of 0.05 having sufficient statistical power to calculate the sample size [92]. Usually, 0.80 Power (1 - β err prob) has been chosen which is suitable for quantitative study [93]. As it’s a moderated mediation model with one independent variable, one mediator, one moderator, and one dependent variable and the analysis has been done on Smart PLS 4, therefore the number of predictors is three because Smart PLS treats the interaction term between mediator and moderator as a single predictor [94]. The calculated minimum sample size is 77 which justifies the sample selection in this study.

Table 1 displays the frequency distribution of the study’s respondents. The majority of respondents, accounting for 78.8% of the total sample, are male. Female respondents make up 21.2% of the total. In terms of age, the largest group falls within the 26–30 age bracket, comprising 31.1% of the total sample, followed by the 36–40 age group, representing 24.5%. Conversely, the smallest group consists of employees aged 46 and above, constituting only 0.4% of the total sample. Regarding work experience, most employees have 6–10 years of experience, accounting for 30.0% of the total, while the second largest group has 0–5 years of experience at 24.3%. The least represented group has 21 or more years of experience, making up 5.1% of the total. In terms of educational qualifications, the majority of employees hold a Bachelor’s degree, making up 34.6% of the respondents, while the second largest group holds a Master’s degree, representing 28.8% of the total respondents.

**Table 1:** Demographics

Gender	Frequency	Percentage
Male	383	78.8%
Female	103	21.2%
**AGE**		
20–25	66	13.6%
26–30	151	31.1%
31–35	115	23.7%
36–40	119	24.5%
41–45	33	6.8%
46-Above	2	0.4%
**EXPERIENCE (Years)**		
0–5	118	24.3%
6–10	146	30.0%
11–15	91	18.7%
16–20	106	21.8%
21-Above	25	5.1%
**Educational Qualification**		
Intermediate	113	23.3
Bachelors	168	34.6
Masters	140	28.8
MS/MPHIL	60	12.3
Others	5	1.0

Source: Developed by the author

### Measurements

The second segment of the survey focuses on organizational virtuousness and includes five items adapted from Cameron, Bright [8] to assess this construct. An example of a construct of OV is “People are treated with courtesy, consideration, and respect in this organization”. The third section elaborates on OCB and encompasses nine items adapted from the research of [95] to measure this particular aspect of employee behavior. One of the items of OCB is “I voluntarily help others who have work-related problems”. Subsequently, the questionnaire addresses workplace ostracism, featuring five items that have been adapted from the work of [35] to evaluate employees’ experiences of exclusion or neglect within the workplace. One of the important items of WO is “Others ignored you at work”. The final part of the questionnaire pertains to employee job performance and incorporates five items adapted from the research conducted by Janssen and Van Yperen [96] to assess this critical aspect of employee contribution. An example of the item is “I (employee) consistently complete the duties specified in my job description”. The shorter questionnaire has been adapted due to the potential of survey fatigue and respondent time limitations to achieve a high number and accurate responses [97]. Adapting the items are relevant to the culture and objectives representing each dimension of variables. The survey reliability and validity of the survey can be improved by using validating questionnaires by reducing time and cross-validation of the results [98]. To maintain the applicability, specificity, and conciseness of the survey, a limited number of items have been chosen. The items have been chosen after a careful study with the relevance of research objectives and design. A focused set of items allows the researcher to align their measurement approach with the hypothesis and specific research objectives [99]. Research suggests that the long questionnaire is difficult to fill by the respondents and can lead to decreased motivation, and response fatigue and can enhance the response errors [100]. The concise set of well-designed items has been selected to prioritize the quality of data based on the factor loading. Moreover, research suggests that a shorter version of the questionnaire helps reduce measurement errors [101].

A questionnaire has been designed on the careful observation of pilot testing and reliability. A pilot study has been done on a small sample size of almost 70 samples. The reason for conducting the pilot testing is the reduction of items of OV and WO. Previously OV had 15 items with 5 dimensions. Further ten items have been used for workplace ostracism and reduced to 5 items. Pilot testing has been done to handle the issues of redundant items and response bias. Most of the items have been deleted due to less understanding of items exhibiting poor performance and less factor loading. Items showing factor loading below 0.7 have been removed in the pilot testing procedure [102–104].

### Structural equation modeling

Following the demographic analysis, the study employed structural equation modeling to analyze the dataset. This analysis included both the measurement model and the path coefficient model. To minimize the problem of common method variance, confidentiality and anonymity have been assured which motivates participants to be more honest and give unbiased responses. Moreover, SEM (Structural Equation Modelling), or method factor loading can also help control the common method variance during data analysis [105]. Therefore in this study, Structural Equation Modelling and measurement models have been used. The basic partial least squares (PLS-SEM) model was utilized to estimate the model, and hypotheses were evaluated for acceptance or rejection. SMART PLS 4 was the chosen tool for data analysis due to its established effectiveness in assessing reliability, validity, and confirming or refuting hypotheses. The most popular choice in analyzing structural equational modeling is Smart PLS (Partial Least Squares) including the moderated mediation models. While other software packages like SPSS also offer capabilities for structural equational modeling, there are different reasons for considering Smart PLS stronger to analyze moderated mediation models. The reason for selecting Smart PLS can be drawn on certain important characteristics specifically for moderated mediation models. Those capabilities and characteristics have been acknowledged in the previous articles and are advantageous for SEM (structural equational modeling) and specifically moderated mediation modeling. Smart PLS is the most important tool for reliability and structural equational modeling. Smart PLS 4 has been selected to analyze measurement model and path analysis because the advanced methodology of Partial Least Square has been used in this study which is particularly for exploratory studies and complex models. Non-normal data and small sample sizes are preferred in Smart PLs [104]. Bootstrapping techniques have been used as a default technique in Smart Pls 4 for default resampling and estimating standard errors, and significant levels of confident intervals. Detailed analysis of the measurement model can be done through reliability, validity, composite reliability, and average variance extracted providing a detailed report for the data [102].

In Smart PLS, there is flexibility in the specification of the model because it allows both formative and reflective measurement models as compared to traditional software like SPSS. Through this flexibility complex constructs can be handled or when the measurement model needs to be modified according to the specific research context [106]. Moreover, non-normal data and small sample sizes can be handled by Smart PLS robustly which makes the analysis suitable for data deviating from the normality and limited sample size. This methodology is specifically important for moderated mediation models in which complex models are being investigated [107].

In most cases, predictive modeling can be analyzed through the Smart PLS through which outcomes can be predicted rather than analyzing the complex theoretical models [104]. Many research contexts related to business and social sciences have been aligned in the Smart PLS. A variance-based approach has been used by Smart PLS which handles multi-collinearity relying on latent variables [108]. This can help handle multi-collinearity which is frequently a concern in the moderated mediation model specifically when there is interaction term analysis.

The initial step involved the measurement model, which aimed to assess the quality of the constructs through evaluations of reliability and validity. Discriminant validity was established using the Heterotrait-Monotrait ratio (HTMT) table. Subsequently, factor loadings were scrutinized to evaluate the strength of the underlying constructs. The table also provided valuable information on Cronbach’s Alpha values and composite reliability. The subsequent phase, the structural model, was employed to explore relationships between variables. This model allowed for the investigation of mediation, moderation, and moderated mediation through the utilization of the bootstrapping method.

#### Analysis of the measurement model

In the measurement model, we assessed discriminant validity based on criteria from prior research, which presented thresholds of 0.85 or 0.90. For our study, we adopted the more stringent criterion of 0.85 [109]. Table 2 presents the results, demonstrating that all values are below 0.85 and thus considered valid for further analysis.

**Table 2:** Discriminant validity

	Constructs	1	2	3	4	5
1	JP					
2	OCB	0.686				
3	OV	0.674	0.798			
4	WO	0.769	0.784	0.694		
5	WO x OV	0.433	0.478	0.540	0.640	

Source: Developed by the author

#### Factor loading and path coefficient

The most crucial aspect of the measurement model involves assessing the factor loading of each item within a construct and determining the overall reliability of each variable. Reliability and validity can be checked by Smart PLS with the most robust procedure, particularly in the field of social sciences and business research. Reliability can be checked through internal consistent reliability and indicator reliability [94]. In this study, internal consistent reliability has been focused on calculating composite reliability for each latent variable. Validity has been checked through convergent validity [102], Discriminant validity, and cross-loading. In this study, convergent validity and discriminant validity have been used with a high loading of ideally higher than 0.7 and Ave more than 0.5. Reliability can be analyzed by factor loading with a value of more than 0.6 which shows the substantial relationship between the latent factor and observed variable showing a meaningful contribution.

The study was done by [38] on the relationship between organizational virtuousness, subjective well-being, and job performance and found a factor loading more than 0.6 supporting the results. In another study by [84] mediated moderation model was studied among workplace spirituality and OCB with the moderation of WO. The study accepted the factor loading of items more than 0.6 reliable.

As presented in Table 3, the factor loading values for each item are scrutinized. Typically, scholars suggest that factor loading values exceeding 0.7 are considered acceptable [110]. However, in this study, any factor loading values below 0.6, particularly within the constructs of organizational virtuousness and workplace ostracism, were removed due to their potential impact on the results. Additionally, this table provides insights into the reliability metrics, including Cronbach’s Alpha, Composite Reliability (CR), and Average Variance Extracted (AVE). The overall reliability of the constructs exceeds 0.7, and the AVE surpasses 0.5, indicating that all items are reliable and valid for further analysis, in line with the recommendations of [111].

**Table 3:** Factor loading and reliability

	Items	Factor Loading	Cronbach’s Alpha	CR	AVE
	OV1	0.866			
	OV2	0.788			
OV	OV3	0.753	0.902	0.902	0.649
	OV4	0.813			
	OV5	0.803			
	JP1	0.743			
	JP2	0.831			
JP	JP3	0.823	0.897	0.897	0.637
	JP4	0.833			
	JP5	0.756			
	OCB1	0.807			
	OCB2	0.777			
OCB	OCB3	0.756			
	OCB4	0.777			
	OCB5	0.749	0.935	0.935	0.614
	OCB6	0.758			
	OCB7	0.768			
	OCB8	0.836			
	OCB9	0.819			
	WO1	0.772			
	WO2	0.823			
WO	WO3	0.873	0.913	0.914	0.679
	WO4	0.851			
	WO5	0.798			

Source: Developed by the author

#### Analysis of structural model

**Fig. 2:**
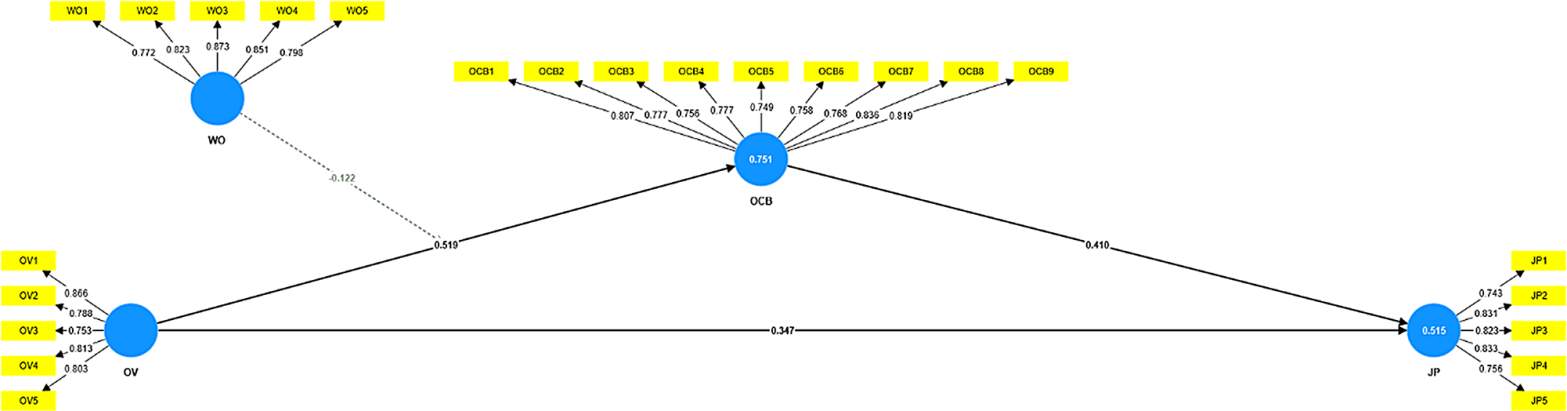
Path coefficient and factor loading. (Source: Developed by the author)

Table 4; Fig. 2 display the structural equation modeling findings. This study examined three hypotheses drawn from the literature review. The first hypothesis looked into the connection between OV and the JP of employees. The results revealed a significant relationship, with a Beta coefficient of 0.347, t-stats of 5.500, and a p-value of 0.000. Consequently, the first hypothesis was accepted, supported by an R-square value of 0.513 and an F-square of 0.090. The second hypothesis looked at how organizational citizenship behavior mediated the association between organizational virtuousness and employee job performance. It also showed a statistically significant relationship, with a Beta coefficient of 0.213, t-stats of 6.005, and a p-value of 0.000, leading to the acceptance of the second hypothesis. As compared to the second hypothesis where the mediation of OCB has been discussed the Beta value is 0.213 which shows partial mediation. The third hypothesis explored the moderating effect of workplace ostracism on the mediation of organizational citizenship behavior in the relationship between organizational virtuousness and job performance. This hypothesis was also accepted, with a significant Beta coefficient of -0.050, t-stats of 2.719, and a p-value of 0.007. The negative Beta coefficient suggests that workplace ostracism weakens the mediation of organizational citizenship behavior in the relationship between organizational virtuousness and employee job performance. When the moderation of WO has been checked on the mediation of OCB, the Beta value which is 0.050 has been shifted to the negative side which shows the negative and significant moderation. Banks have observed less OCB and hence employee job performance with the negative role of workplace ostracism. Employees working in a collective culture are deeply affected by the ostracized practices in the organization which shows the implications of interventions in the HR practices to solve this problem. Previously the mediated moderation model has been checked regarding workplace spirituality with the mediation of organizational commitment and moderation of workplace ostracism [84]. All hypotheses have been accepted and negative moderation of workplace ostracism has been observed similar to this study. This analysis revealed a negative moderation effect on the relationship between organizational virtuousness and job performance.

**Table 4:** Summary of results

Hypothesis	Standard Beta	t-Stats	*p*-Value	*R* ^2^	F^2^	Results
OV -> JP _(H1)	0.347	5.500	0.000	0.513	0.090	Supported
OV -> OCB	0.519	9.018	0.000	0.749	0.538	Supported
OCB -> JP	0.410	6.719	0.000	0.513	0.125	Supported
WO -> OCB	-0.524	9.793	0.000	0.749	0.436	Supported
WO x OV -> OCB	-0.122	3.042	0.002	0.749	0.044	Supported
OV -> OCB -> JP (H2)	0.213	6.005	0.000			Supported
WO -> OCB -> JP	-0.215	5.000	0.000			Supported
WOxOV-> OCB-> JP(H3)	-0.050	2.719	0.007			Supported

Source: Developed by the author

## Conclusion and discussion

In a developing country like Pakistan, the banking sector plays a pivotal role in boosting the economy, making financial services accessible to more people, and contributing to the nation’s overall progress. However, in recent times especially during the COVID-19 epidemic, the sector faced significant challenges in maintaining trust among its customers and stakeholders. These challenges include issues like fraud, cybersecurity threats, and economic instability, which harmed the sector’s reputation. Alongside regulatory constraints and economic instability, intense competition exerted additional pressure on the sector’s profitability and its efforts to achieve consistent growth in Pakistan. In light of these challenges, this study investigated the factors that can enhance bank performance. Given that organizational performance depends on employee performance, this study investigates the influence of organizational virtuousness on employee job performance. Additionally, the study explores the mediating role of organizational citizenship behavior in the relationship between organizational virtuousness and employee job performance, as well as the moderating effect of workplace ostracism.

To achieve these objectives, we collected questionnaire-based data from employees working in various banks in Pakistan, resulting in a final sample of 486 responses. The study tested the above hypothesis using structural equation modeling. The results provided strong support for the first hypothesis, showing that OV is statistically significantly and positively associated with job performance. Second, our results support the second hypothesis by confirming the important mediating role that organizational citizenship behavior plays in the link between organizational virtuousness and employee job performance. Finally, we found a negative moderating effect of workplace ostracism in the mediation of organizational citizenship behavior on the relationship between organizational virtuousness and job performance. Therefore, the results also support the third hypothesis of the study. Employees with positive perceptions are satisfied and happy and make more robust relationships with co-workers and the work [112] and hence show good performance.

Workplace ostracism is the main issue that can reduce employee performance and citizenship behavior. The results above supported the view that organizational virtuousness positively affects employee job performance. The association between organizational virtuousness and job performance is significantly mediated by organizational citizenship behavior. Workplace ostracism is the only negative thing impacting employee citizenship behavior and performance, which should be reduced or finished in the organizations. The negative outcomes like employee job performance and organizational citizenship behavior of workplace ostracism have also been elaborated on in the previous research [18], and the findings of this research show the negative moderation of workplace ostracism in the mediation of organizational citizenship behavior in the relationship of organizational virtuousness and job performance.

### Theoretical and practical implications

The findings of this study hold significant managerial and practical implications for organizations aiming to enhance employee job performance. The implementations of these findings must be addressed according to ethical considerations respecting the rights and well-being of employees in the promotion of a positive and sustainable work environment. Firstly, it underscores the importance of cultivating organizational virtuousness within the workplace, emphasizing the need for ethical conduct, teamwork, and employee well-being. Ethically improving job performance metrics is not only the responsibility but also an enhancement in the overall work-life quality of employees. Secondly, the study highlights the pivotal role of organizational citizenship behavior as a mediator in the relationship between organizational virtuousness and job performance, emphasizing the value of encouraging behaviors that go beyond formal job descriptions. Ethically, organizations should encourage and recognize positive employee behaviors without exploiting them. Additionally, the study alerts managers to the detrimental impact of workplace ostracism, revealing its negative moderating effect on the mediation of organizational citizenship behavior. This stresses the urgency of addressing and mitigating instances of ostracism to maximize the positive effects of virtuous behavior. Ethically organizations should be vigilant in observing employees and should ensure that no employee facing ostracism should be left out. From a theoretical standpoint, the research contributes to the growing body of knowledge on organizational behavior, particularly in its nuanced exploration of mediating and moderating effects. Social exchange theory says that when employees positively perceive virtuousness, they will show citizenship behavior towards the organization and good performance.

Unethical practices will reduce employee performance and damage the organization’s image and reputation. CEO of banks has to show the determination to implement virtuous practices in all banks and branches with the help of branch managers. Regular feedback from employees should be taken to ensure the implementation of virtuous practices and avoid workplace ostracism.

Some organizational interventions have to be implemented to show the importance of leadership training in avoiding bullying and harassment which also includes ostracism. The most important intervention is adopting a mechanism of conflict resolution. In the research, the importance of the mechanism of conflict resolution has been highlighted to reduce the tension in the workplace and collaboration promotion [113].

When there is a negative impact of workplace ostracism on the relationship of OV, JP, and OCV, several limitations and alternative explanations should also be considered. There are cultural differences in the organizations of different regions. In some countries, organizations have conformity and hierarchical structures which may lead employees to accept ostracism as a normative behavior diminishing the impact of organizational virtuousness. Cultural values and national culture play a vital role in the determination of consequences of workplace ostracism and its boundary conditions [75]. Some of the important factors that should also be considered are personality traits and cognitive biases. Workers with high levels of self-efficacy and resilience may be less affected by the ostracized practices and vice versa. Overall organizational climate including, communication patterns, organizational norms, and leadership style can reduce the impact and prevalence of workplace ostracism. Moreover, the support from top management can also be helpful to reduce ostracism. Top management support is necessary for the development of the team culture and collective efforts with immediate training [114] to avoid ostracism at the workplace. Individuals can have different coping mechanisms to handle workplace ostracism which may be the social support or withdrawal from organizational activities. These coping mechanisms can influence the impact of ostracism on performance and citizenship behavior.

### Limitations and recommendations for future research

As with any other study, our research also has some limitations that should be acknowledged. Firstly, our study focused on data collected from the banking sector in Pakistan, a collectivist society. This context-specific approach may limit the generalizability of our findings to different cultural and industry settings. Therefore, future research should aim to replicate the study in various cultural and industrial contexts to determine the extent to which our results hold across diverse environments. Secondly, our study employed a cross-sectional research design, which provides a static snapshot of the relationships between variables at a single point in time. To gain a deeper understanding of the temporal dynamics and causal relationships between organizational virtuousness, organizational citizenship behavior, workplace ostracism, and job performance, future research should consider implementing longitudinal studies or experimental designs. Such investigations will contribute to a more comprehensive understanding of these important workplace dynamics and offer actionable insights for organizations seeking to optimize employee performance. Moreover investigating the influence of workplace ostracism on organizational outcomes can be challenging due to the potential biases and subjective nature of the construct. Self-reported measures may be susceptible to memory distortions or social desirability affecting the result validity. Observational data and peer ratings can be done in future research which can be a new methodology in the management sciences research which may be difficult logistically.

### Electronic supplementary material

Below is the link to the electronic supplementary material.

Supplementary Material 1

## Data Availability

Taken into account the anonymity of the participant and the datasets is being used in other unpublished studies, the datasets for this article are not publicly available. However, if it is reasonable requested, please contact the corresponding author to obtain our datasets.

## Abbreviations

OV: Organizational Virtuousness
OCB: Organizational Citizenship Behavior
JP: Job Performance
WO: Workplace Ostracism
SET: Social Exchange Theory
AVE: Average Variance Extracted
