# Supplementary material for: “Exploring the relationship of organizational virtuousness, citizenship behavior, job performance, and combatting ostracism” through structural equational modeling
Source: BMC Psychol. 2024 Jul 9;12:384. doi: 10.1186/s40359-024-01873-9 (PMC11232238; doi:10.1186/s40359-024-01873-9)
Supplement: Supplementary file 1 — Supplementary Material 1 [file 40359_2024_1873_MOESM1_ESM.docx]

**Respected Respondent,**

Hello. I am a PhD scholar in management sciences and engineering. I am researching organizational virtuousness on Job performance with work engagement as a mediator and workplace ostracism as a moderator. You are requested to respond voluntarily and will be appreciated, and your identity will be kept confidential.

Regards

**DEMOGRAPHICS**

| **Gender**   1. Male 2. Female | **Experience**   1. 0-5 2. 6-10 3. 11-15 4. 16-20 5. 21-above |
| --- | --- |
| **Age**   1. 20-25 2. 26-30 3. 31-35 4. 36-40 5. 41-45 6. 46-above | **Education**   1. Intermediate 2. Bachelor’s 3. Master’s 4. Mphil/MS 5. Others  Please specify (…….) |

| **Sr** | **Tick In the Relevant Box** | **Strongly Agree** | **Agree** | **Somewhat Agree** | **Neutral** | **Somewhat Disagree** | **Disagree** | **Strongly Disagree** |
| --- | --- | --- | --- | --- | --- | --- | --- | --- |
|  | **Organizational Virtuousness (Cameron et al., 2004)** |  |  |  |  |  |  |  |
|  | In this organization we are dedicated to doing good in addition to doing well. | 7 | 6 | 5 | 4 | 3 | 2 | 1 |
|  | People are treated with courtesy, consideration, and respect in this organization. | 7 | 6 | 5 | 4 | 3 | 2 | 1 |
|  | This organization is characterized by many acts of concern and caring for other people. | 7 | 6 | 5 | 4 | 3 | 2 | 1 |
|  | This organization demonstrates the highest levels of integrity. | 7 | 6 | 5 | 4 | 3 | 2 | 1 |
|  | This is a forgiving, compassionate organization in which to work. | 7 | 6 | 5 | 4 | 3 | 2 | 1 |
|  | **Organizational Citizenship Behavior (Konovsky and Organ, 1996)** | **Strongly Agree** | **Agree** | **Somewhat Agree** | **Neutral** | **Somewhat Disagree** | **Disagree** | **Strongly Disagree** |
|  | I voluntarily help others who have work-related problems. | 7 | 6 | 5 | 4 | 3 | 2 | 1 |
|  | I always take care to do a thorough job. | 7 | 6 | 5 | 4 | 3 | 2 | 1 |
|  | I don't complain about trivial matters at work. | 7 | 6 | 5 | 4 | 3 | 2 | 1 |
|  | I regularly make an effort to help coworkers who have heavy workloads. | 7 | 6 | 5 | 4 | 3 | 2 | 1 |
|  | I take an active part in activities that help the organization. | 7 | 6 | 5 | 4 | 3 | 2 | 1 |
|  | I often think of new ways to improve things at work. | 7 | 6 | 5 | 4 | 3 | 2 | 1 |
|  | I am often in a good mood at work. | 7 | 6 | 5 | 4 | 3 | 2 | 1 |
|  | I never take supplies home from work that are meant to stay in the office. | 7 | 6 | 5 | 4 | 3 | 2 | 1 |
|  | I'm willing to help others who have heavy workloads, even if I'm busy. | 7 | 6 | 5 | 4 | 3 | 2 | 1 |
|  | **Workplace Ostracism (Ferris et al., 2008)** |  |  |  |  |  |  |  |
|  | Others ignored you at work. | 7 | 6 | 5 | 4 | 3 | 2 | 1 |
|  | Others left the area when you entered. | 7 | 6 | 5 | 4 | 3 | 2 | 1 |
|  | Your greetings have gone unanswered at work. | 7 | 6 | 5 | 4 | 3 | 2 | 1 |
|  | Others at work shut you out of the conversation. | 7 | 6 | 5 | 4 | 3 | 2 | 1 |
|  | Others at work did not invite you or ask you if you wanted anything when they went out for a coffee break. | 7 | 6 | 5 | 4 | 3 | 2 | 1 |
|  | **Job Performance (Janssen and Van Yperen, 2004)** |  |  |  |  |  |  |  |
|  | I (employee) consistently complete the duties specified in my job description. | 7 | 6 | 5 | 4 | 3 | 2 | 1 |
|  | I (employee) consistently meet the performance requirements of the job. | 7 | 6 | 5 | 4 | 3 | 2 | 1 |
|  | I (employee) fulfill all responsibilities required by my job. | 7 | 6 | 5 | 4 | 3 | 2 | 1 |
|  | I (employee) consistently fulfill my obligations to perform for my job. | 7 | 6 | 5 | 4 | 3 | 2 | 1 |
|  | I (employee) often fail to perform essential duties. | 7 | 6 | 5 | 4 | 3 | 2 | 1 |

**References**

CAMERON, K. S., BRIGHT, D. & CAZA, A. (2004). Exploring the relationships between organizational virtuousness and performance. *American behavioral scientist,* 47**,** 766-790.

FERRIS, D. L., BROWN, D. J., BERRY, J. W. & LIAN, H. (2008). The development and validation of the Workplace Ostracism Scale. *Journal of applied psychology,* 93**,** 1348.

JANSSEN, O. & VAN YPEREN, N. W. (2004). Employees' goal orientations, the quality of leader-member exchange, and the outcomes of job performance and job satisfaction. *Academy of management journal,* 47**,** 368-384.

KONOVSKY, M. A. & ORGAN, D. W. (1996). Dispositional and contextual determinants of organizational citizenship behavior. *Journal of organizational behavior,* 17**,** 253-266.
